# Integrating network toxicology and in vitro validation to elucidate PET microplastic-induced osteoarthritis pathogenesis

**DOI:** 10.3389/fphar.2026.1859004

**Published:** 2026-06-30

**Authors:** Zhengtian Li, Lin Wang, Haiquan Huang, Daoyun Lu, Tingting Han, Jinmin Zhao, Gang Du

**Affiliations:** 1 Department of Orthopedic and Trauma Surgery, The First Affiliated Hospital of Guangxi Medical University, Nanning, Guangxi, China; 2 Department of Bone and Joint Surgery, The First Affiliated Hospital of Guangxi Medical University, Nanning, Guangxi, China; 3 Department of Sports Medicine, Eastern Theater Navy Hospital, Zhoushan, China; 4 Department of Stomatology, Weifang Nursing Vocational College, Qingzhou, China

**Keywords:** microplastics, molecular docking, network toxicology, osteoarthritis, WGCNA

## Abstract

**Objective:**

This study aims to elucidate the molecular mechanisms through which PET microplastics (PET-MP) influence osteoarthritis (OA) pathogenesis by integrating network toxicology, machine learning, and *in vitro* experimental validation.

**Methods:**

Differential gene expression analysis and WGCNA were applied to multiple OA datasets to identify disease-related targets. PET-MP biological targets were predicted via ChEMBL, SwissTargetPrediction, and PharmMapper. Overlapping targets were screened using machine learning algorithms, and molecular docking was performed to assess binding interactions. *In vitro* validation including immunofluorescence, qRT-PCR, and Western blot was conducted in PET-MP-treated chondrocytes.

**Results:**

A total of 452 PET-associated targets were identified, with 12 core PET-MP-OA genes established through intersection analysis. Functional enrichment implicated the NF-κB and IL-17 signaling pathways. Machine learning screening based on feature importance and SHAP values prioritized six hub genes: AKR1A1, INSR, KIF11, MMP1, KCNN4, and TK1. Molecular docking generated predicted AutoDock Vina scores ranging from −3.893 to −7.434 kcal/mol. *In vitro* experiments validated upregulation of AKR1A1, MMP1, KCNN4, KIF11, and TK1, and downregulation of INSR in chondrocytes, consistent with bioinformatics predictions.

**Conclusion:**

PET-MP may promote OA progression by disrupting molecular pathways related to inflammation, oxidative stress, and cartilage degradation. The identified hub genes offer new insights into microplastic toxicology in joint disease and represent potential therapeutic targets and biomarkers for PET-MP-induced OA.

## 1 Introduction

Over 500 million people throughout the world are affected by osteoarthritis (OA), which is the most prevalent form of chronic joint disease. It accounts for a substantial proportion of the global burden of disability, contributing significantly to disability-adjusted life-years (DALYs) (Neogi and Colloca, 2023; Mahmoudian et al., 2021). The progressive loss of articular cartilage, subchondral bone sclerosis, osteophyte formation, and chronic synovial inflammation are the hallmarks of OA. Pain, stiffness, and a loss in functional ability are the results of these alterations (Katz et al., 2021). Conventional risk factors, which include age, gender, genetics, obesity, and mechanical damage, are unable to adequately explain the rapid increase in incidence that has been observed in metropolitan regions. As a result, environmental factors have been the subject of a significant amount of investigation (Winiarska et al., 2024; Vethaak and Legler, 2021; Qin et al., 2024). Plastics have a tremendous amount of applications all over the world because of their exceptional qualities, which include their affordability and their durability. Plastics are utilized extensively all over the world as a result of their excellent features, which include their long-lasting nature and their cost-effectiveness. Microplastics, often known as MPs, are defined as plastic particles that have a diameter of significantly less than 5 millimeters (Wang et al., 2021). One of the most common types of microplastics is known as polyethylene terephthalate microplastics, or PET-MP for short. Because of their beneficial qualities, such as their lightweight, moldability, and cost-effectiveness, PET-MP comprise a large component of the manufacturing of plastic. They are frequently utilized in the packaging of food and beverages (Pencik et al., 2023). As a consequence of the widespread manufacture of PET microplastics, their presence can be found in virtually every aspect of daily life. According to the findings of research, PET-MP and other pollutants of a similar kind can be found in marine and river waters, sediments, aquatic species, dust, and even consumables like sea salt, drinks, bottled water, and drinking water. It has also been discovered that PET-MP can be found in human feces, blood, and lung tissues (Menon et al., 2023; Sun et al., 2025). The presence of micro- and nanoplastics, which have been highlighted as new concerns, has been discovered in human blood, lung tissue, liver tissue, and placental tissue; however, the involvement of these substances in joint pathology has not yet been well investigated. Although there has not been sufficient research conducted on the potential impact of PET-MP on OA, similar studies have suggested that environmental pollutants may disrupt the homeostasis of individual joints (Liu et al., 2018; Skalny et al., 2024). Based on research conducted on a number of pollutants, it has been proven that these pollutants have the ability to induce oxidative stress, inflammatory reactions, and abnormal cellular communication in joint tissues. These are all essential factors in the progression of OA (Zhu et al., 2024). Considering the ubiquitous occurrence of PET-MP and the rising incidence of OA, it is essential to examine the correlation between exposure to PET-MP and the pathogenesis of OA.

PET-MP is a major secondary plastic component derived from beverage bottles, textile microfibers, and food-contact polymers. Studies have found that microplastics can accelerate the progression of OA by inducing and accelerating inflammatory responses (Park et al., 2013; Rawle et al., 2022).Following internalization by articular chondrocytes and synoviocytes, PET-MP particles initiate a multistep pathogenic cascade resulting in OA-like joint destruction. Surface-reactive oxygen species (ROS) generated at the PET-MP interface exceed the capacity of cellular antioxidant defenses, leading to sustained oxidative stress and lipid peroxidation. Mitochondrial membrane depolarization results in decreased ATP production and activates the NLRP3 inflammasome, subsequently facilitating the caspase-1-dependent maturation of IL-1β and IL-18 (Grässel et al., 2021). The cytokines subsequently enhance the expression of MMP-13 and ADAMTS-5, resulting in the degradation of type-II collagen and aggrecan, respectively (Xia et al., 2014). IL-1β–induced ROS simultaneously stabilize nuclear β-catenin, promoting chondrocyte hypertrophy and the expression of COL10A1 and RUNX2, thereby accelerating cartilage calcification (Valdes et al., 2012). The molecular events outlined correspond to the oxidative, metabolic, and inflammatory features observed in early OA lesions, indicating that PET-MP may represent a previously underestimated factor in joint degeneration.

OA arises from a disruption in the synthesis and degradation of the extracellular matrix, affected by chondrocyte hypertrophy, oxidative stress, and ongoing low-grade synovial inflammation. Furthermore, both biomechanical overload and genetic susceptibility can trigger the Wnt/β-catenin, NF-κB, and ADAMTS-5 signaling pathways. This activation leads to the breakdown of articular cartilage, the hardening of subchondral bone, and eventually, joint dysfunction (He et al., 2020). PET-MP have been shown to aggravate pathways in extra-articular tissues, such as ROS-mediated lipid peroxidation, ER stress, and DNA damage responses, ultimately resulting in apoptosis or senescence (Wang et al., 2023; Wang et al., 2022; Herrala et al., 2023). The functioning of analogous mechanisms in cartilage and synovium, along with the molecular responses of intracellular targets associated with PET-MP exposure, remains to be systematically examined. This research combines network toxicology, machine learning, and molecular docking to examine the transcriptomic and structural basis of OA induced by PET-MP.

Recent advancements in network toxicology provide a systems-level framework for delineating xenobiotic–host interactions by integrating high-throughput omics, chemical–protein interaction databases, and machine-learning-based feature selection. This paradigm has successfully identified druggable hubs in aflatoxin-induced hepatocellular carcinoma and doxorubicin cardiotoxicity; however, its application in musculoskeletal research is limited. We hypothesized that PET-MP interfere with a specific molecular network in chondrocytes and synoviocytes. By prioritizing targets within this network and conducting exploratory molecular docking analysis, we aim to identify viable therapeutic targets.

This study comprehensively examines the impact of PET-MP on OA through advanced bioinformatics and molecular biology methodologies. Our objective is to offer an all-encompassing comprehension of the molecular mechanisms that underlie the effects induced by PET-MP on OA. This will be achieved by adhering to a systematic process, starting from target identification and proceeding to molecular docking.This approach may facilitate future research and interventions in the emerging field of environmental musculoskeletal health, and we further validate our computational findings through *in vitro* experimental approaches including immunofluorescence, qRT-PCR, and Western blot analysis in PET-MP-treated chondrocytes.

## 2 Materials and methods

### 2.1 Acquisition of disease-related targets

**FIGURE 1:**
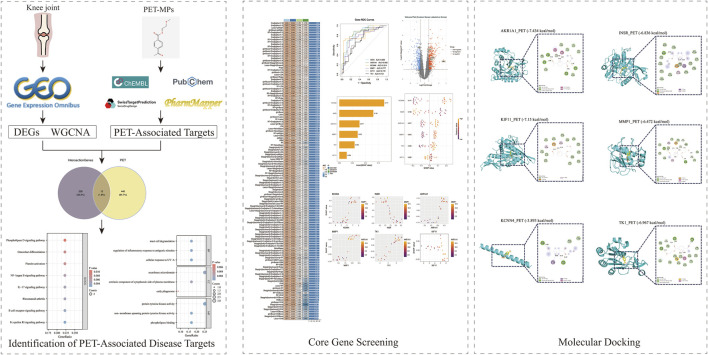
Flow-chart of datasets analysis in this paper.

Six transcriptomic datasets (GSE55235, GSE55457, GSE82107, GSE12021, GSE32317, and GSE55584) were obtained from the NCBI GEO database. The discovery cohort included GSE55235, GSE55457, and GSE82107, whereas the validation cohort consisted of GSE12021, GSE32317, and GSE55584. A multi-stage normalization pipeline was implemented to mitigate batch effects. Surrogate Variable Analysis (SVA) is a statistical method used to identify and account for latent variables that may confound the relationship between observed variables in high-dimensional data. This approach enhances the accuracy of inference by adjusting for hidden sources of variation, thereby improving the robustness of the results. Latent confounding variables within the discovery cohort were modeled and adjusted through the application of the SVA package. For ComBat Harmonization, residual batch effects were rectified utilizing parametric empirical Bayes frameworks. Post-correction principal component analysis (PCA) showed improved clustering of samples across different batches in the reduced-dimensional space, which thus confirmed the effectiveness of data harmonization. The complete analytical workflow is depicted in Figure 1.

### 2.2 Acquisition of chemical components and targets of PET

PET was characterized through the integration of data from multiple databases. The canonical 2D structural descriptors of PET (SMILES notation: CC(=O)C1=CC=C(C=C1)C(=O)OCCOC) and its physicochemical properties were retrieved from the PubChem database (https://pubchem.ncbi.nlm.nih.gov/). A three-pronged strategy was adopted for target prediction: the ChEMBL Database was used for profiling ligand-receptor interactions (https://www.ebi.ac.uk/chembl/); SwissTargetPrediction served for chemical genomics-based forecasting (http://www.swisstargetprediction.ch); and PharmMapper was applied for three-dimensional pharmacophore matching (http://lilab-ecust.cn/pharmapper). All predicted targets were restricted to the *Homo sapiens* proteome.

### 2.3 Differential gene expression analysis

The limma package was employed to assess the transcriptome data. Differentially expressed genes (DEGs) were detected with the criteria of FDR-adjusted P < 0.05 and |log2FC| > 0.585, which is equivalent to a 1.5-fold change. The results were visualized using ggplot2.

### 2.4 Weighted gene co-expression network analysis (WGCNA)

A weighted gene co-expression network was constructed using the WGCNA R package. The expression matrix was normalized, and genes with low variance were removed before network construction. Sample and gene quality were assessed using goodSamplesGenes(), and potential sample outliers were evaluated by hierarchical clustering. The soft-thresholding power (β) was selected using pickSoftThreshold across candidate powers from 1 to 20 according to the scale-free topology criterion. An unsigned weighted adjacency matrix was then constructed using the selected β, followed by calculation of the topological overlap matrix (TOM). Gene modules were identified by hierarchical clustering of TOM-based dissimilarity followed by dynamic tree cutting, with deepSplit = 2, pamRespectsDendro = FALSE, and a minimum module size of 50 genes. Similar modules were merged based on module eigengene correlation using a merge cut height of 0.30. Module–trait associations were assessed by Pearson correlation between module eigengenes and clinical phenotypes, with P values calculated using corPvalueStudent. Candidate hub genes within disease-associated modules were prioritized according to module membership (kME).

### 2.5 Identification of PET-Associated disease targets

Candidate PET-associated OA targets were identified using a stepwise intersection workflow. First, OA-associated genes were defined by intersecting the DEGs obtained from limma analysis with the OA-associated WGCNA module genes. The numbers of genes in each input set, their overlap, and the calculation basis for the Venn diagram percentages were recorded. Second, the resulting OA-associated overlapping genes were intersected with computationally predicted PET targets from ChEMBL, SwissTargetPrediction, and PharmMapper to obtain PET-associated OA candidate targets for subsequent machine-learning screening. The overlapping relationships were visualized using Venn diagrams.

### 2.6 Functional enrichment analysis

Functional enrichment analysis was performed using the clusterProfiler R package. Gene Ontology (GO) enrichment analysis, including biological process (BP), cellular component (CC), and molecular function (MF) categories, and Kyoto Encyclopedia of Genes and Genomes (KEGG) pathway enrichment analysis were conducted to annotate the biological functions and pathway associations of the candidate genes. Terms or pathways with P < 0.05 were considered statistically significant.

### 2.7 Machine learning-based core gene screening

Machine-learning analysis was performed as a candidate-gene prioritization step using the 12 PET-associated OA candidate genes obtained from the preceding DEG/WGCNA/PET-target intersection workflow. The training cohort consisted of 57 samples from GSE55235, GSE55457, and GSE82107, including 30 OA samples and 27 controls. The external validation cohort consisted of 54 samples from GSE12021, GSE32317, and GSE55584, including 25 OA samples and 29 controls. A total of 127 algorithm, parameter, and two-stage feature-selection/modeling combinations were evaluated, including Lasso, Ridge, Elastic Net, SVM, Random Forest, glmBoost, Stepwise GLM, GBM, LDA, XGBoost, plsRglm, and Naive Bayes-based combinations. Model performance was assessed using ROC analysis and AUC values in the training cohort and in each external validation dataset.

### 2.8 Model interpretation

To improve the interpretability of the machine-learning results, SHAP (SHapley Additive exPlanations) analysis was performed to estimate the contribution of each candidate gene to model predictions. SHAP values were used to quantify the direction and relative magnitude of each gene’s influence on the predicted classification outcome.

### 2.9 Molecular docking analysis

To explore the potential binding poses between PET and the identified core proteins, molecular docking simulations were performed. The molecular structure of PET was obtained from the PubChem database, and the experimentally determined crystal structures of the core proteins were obtained from the Research Collaboratory for Structural Bioinformatics Protein Data Bank (RCSB PDB). The receptor structures were prepared and converted into PDBQT format, and docking was performed using AutoDock Vina. AutoDock Vina scores were interpreted as computational docking scores rather than experimentally measured binding free energies. Docking poses and interaction patterns were analyzed and visualized using Discovery Studio 2019. The top-ranked docking pose of each PET-protein complex was further analyzed to identify residue-level interaction patterns, including hydrogen-bond contacts, carbon hydrogen bonds, van der Waals contacts, π-related interactions, and electrostatic interactions. Detailed PDB identifiers, structural quality metrics, grid-box coordinates, and docking parameters are provided in Supplementary Table S1, and residue-level interaction profiles are provided in Supplementary Table S2.

### 2.10 Cell culture and treatment

Human chondrocytes were used for *in vitro* validation experiments. Cells were maintained in Dulbecco’s Modified Eagle Medium (DMEM) supplemented with 10% (v/v) fetal bovine serum (FBS) and 1% (v/v) penicillin-streptomycin at 37 °C in a humidified atmosphere containing 5% CO2. Cells were subcultured every 48-72 h using 0.125% trypsin-EDTA, and all experiments were conducted using cells that had undergone no more than two passages. Polyethylene terephthalate microplastic (PET-MP) microspheres (diameter approximately 5 μm; solid content 1% w/v, equivalent to a 10 g/L stock concentration) were purchased from Beijing Zhongke Keyou Technology Co., Ltd. (Beijing, China) and stored under light-protected conditions at 2 °C-8 °C. The final working concentration of PET-MPs was 2.4 μg/mL, selected to approximate the reported upper PET-specific concentration measured in human whole blood by Leslie et al. (2022). This concentration is also consistent with a recent OA/RA-related PET-MP co-culture study using the same exposure level (Di et al., 2025).

Lipopolysaccharide (LPS, from *Escherichia coli* 0111:B4; Sigma-Aldrich, St. Louis, MO, USA) was used as an inflammatory stimulus at a final concentration of 1 μg/mL. Cells were divided into three experimental groups: G1, untreated control chondrocytes; G2, chondrocytes co-treated with LPS (1 μg/mL) and PET-MPs (2.4 μg/mL) for 24 h; and G3, chondrocytes treated with LPS (1 μg/mL) alone for 24 h. This three-group design was used to distinguish PET-MP-associated effects under inflammatory conditions from LPS-induced responses alone. All treatments were performed in at least three independent biological replicates.

### 2.11 Immunofluorescence staining

Chondrocytes from the three experimental groups were fixed with 4% paraformaldehyde for 15 min, permeabilized with 0.1% Triton X-100 for 10 min, and blocked with 5% bovine serum albumin (BSA) for 1 h at room temperature. Cells were then incubated overnight at 4 °C with primary antibodies against AKR1A1, MMP1, KCNN4, KIF11, TK1, and INSR. After washing with PBS, cells were incubated with fluorescence-conjugated secondary antibodies for 1 h at room temperature in the dark. Nuclei were counterstained with DAPI. Fluorescence images were captured using a fluorescence microscope, and fluorescence intensity was quantified using ImageJ software.

### 2.12 Quantitative real-time PCR (qRT-PCR)

Total RNA was extracted from chondrocytes in each group using TRIzol reagent. Complementary DNA (cDNA) was synthesized from 1 μg of total RNA using a reverse transcription kit according to the manufacturer’s instructions. qRT-PCR was performed using SYBR Green Master Mix on a real-time PCR system. The relative mRNA expression levels of AKR1A1, MMP1, KCNN4, KIF11, TK1, and INSR were calculated using the 2^−ΔΔCT^ method, with GAPDH serving as the internal reference gene. The forward and reverse primer sequences, expected amplicon lengths, and annealing temperatures are provided in Supplementary Table S3.

### 2.13 Western blot analysis

Total protein was extracted from chondrocytes of each group using RIPA lysis buffer supplemented with protease and phosphatase inhibitors. Protein concentrations were determined by the BCA assay. Equal amounts of protein (30 μg) were separated on SDS-PAGE gels and transferred onto PVDF membranes. Membranes were blocked with 5% non-fat milk for 1 h, then incubated overnight at 4 °C with primary antibodies against TK1 (27 kDa), INSR (100 kDa), KCNN4 (48 kDa), MMP1 (50 kDa), and GAPDH (36 kDa) as the loading control. After washing, membranes were incubated with HRP-conjugated secondary antibodies for 1 h at room temperature. Protein bands were detected using an enhanced chemiluminescence (ECL) detection system, and band intensities were quantified using ImageJ software. Relative protein levels were normalized to GAPDH expression. Detailed antibody information, including host species, dilution ratios, manufacturers, and catalog numbers for all primary and secondary antibodies, is provided in Supplementary Table S4.

### 2.14 Statistical analysis

All experimental data are presented as mean ± standard deviation (SD) from at least three independent experiments. Statistical comparisons among multiple groups were performed using one-way analysis of variance (ANOVA) analysis. A P-value less than 0.05 was considered statistically significant. All statistical analyses were performed using GraphPad Prism software.

## 3 Results

### 3.1 Identification of PET target proteins

**FIGURE 2:**
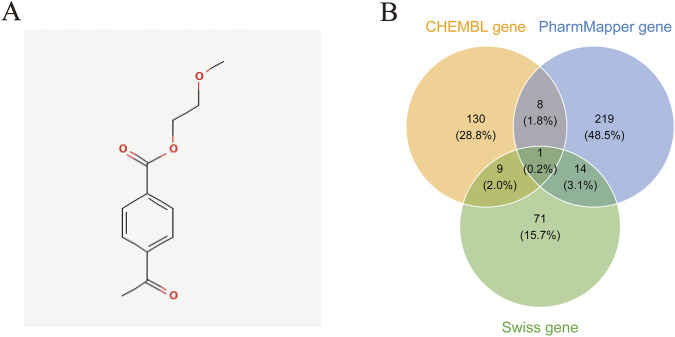
**I**dentification of polyethylene terephthalate microplastics (PET-MP) Target Proteins. **(A)** Chemical structure of PET-MP. **(B)** Potential biological targets were predicted using three complementary computational approaches: CHEMBL, PharmMapper, and SwissTargetPrediction.

Figure 2A displays the molecular configuration of PET, as sourced from the PubChem repository. To systematically predict its putative biological targets, we employed three complementary computational approaches: ChEMBL (for annotated bioactive molecules), PharmMapper (based on reverse pharmacophore matching), and SwissTargetPrediction (which utilizes ligand similarity for target inference). A total of 452 distinct possible targets were found following data integration and the elimination of duplicate entries (Figure 2B).

### 3.2 Identification of OA-Related Target Genes

**FIGURE 3:**
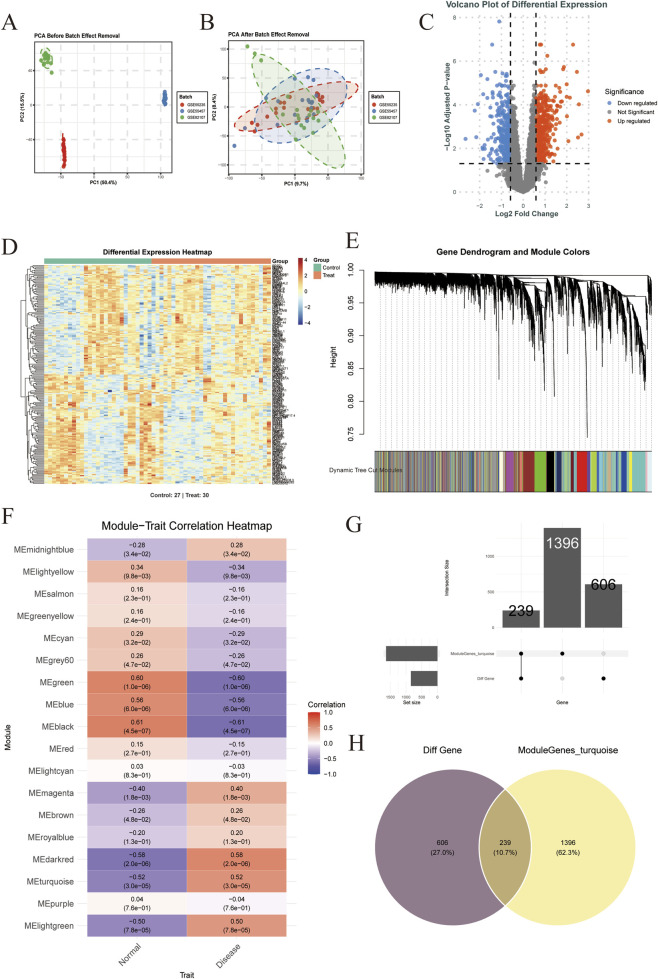
Identification of OA-Related Target Genes. **(A)** PCA scatter plot shows distinct separation between GSE55235, GSE82107 and GSE55457 datasets before batch correction, indicating batch effects. **(B)** PCA scatter plot after batch correction shows the integration of GSE55235, GSE82107 and GSE55457 datasets, indicating reduced batch effects. **(C)** The volcano plot visualizes differentially expressed genes (DEGs), with the x-axis representing the log2 fold change (logFC) and the y-axis corresponding to the statistical significance level (-log10 adjusted p-value). Genes exhibiting significant upregulation are depicted as red data points, whereas those significantly downregulated are shown in blue. Genes without statistically significant changes are indicated in grey. **(D)** The heatmap illustrates the expression profiles of differentially expressed genes (DEGs) among the samples, where red color denotes upregulated expression and blue represents downregulated expression. **(E)** The gene dendrogram generated by WGCNA displays hierarchical clustering of genes according to their co-expression relationships. Distinct color assignments in the lower section correspond to individual gene modules identified through this analysis. **(F)** Module–Trait Association Heatmap: A heatmap illustrates the correlations between gene co-expression modules derived from WGCNA and specific sample traits (Normal vs. Disease). Numerical values within each cell denote the correlation strength and associated statistical significance (p-value). **(G)** Venn diagram shows DEGs and WGCNA modules.

To mitigate technical variation among datasets, the gene expression datasets GSE55235, GSE55457, and GSE82107 were merged and normalized. PCA showed improved sample distribution after batch correction and normalization, indicating reduced dataset-related variation in the integrated expression matrix (Figure 3A,B). Differential expression analysis identified 845 DEGs associated with OA, which were visualized using volcano plots and heatmaps (Figure 3C,D).

WGCNA was then performed to identify OA-associated co-expression modules. After sample and gene quality control, the soft-thresholding power was selected using the scale-free topology criterion across candidate powers from 1 to 20. A soft-thresholding power of β = 5 was selected for network construction. Using this parameter, an unsigned weighted adjacency matrix and TOM-based dissimilarity matrix were generated. Hierarchical clustering followed by dynamic tree cutting identified co-expression modules with a minimum module size of 50 genes. Similar modules were subsequently merged using a merge cut height of 0.30. This analysis identified 18 co-expression modules, each represented by a distinct color (Figure 3E). Module–trait correlation analysis identified the turquoise module as the OA-associated module used for downstream screening (Figure 3F). This module contained 1,635 genes.

Intersecting the 845 DEGs with the 1,635 turquoise-module genes yielded 239 overlapping OA-associated genes (Figure 3G,H). The Venn percentage of 10.7% was calculated using the union of the two input sets as the denominator: 239/(845 + 1,635 − 239) = 239/2,241 = 10.7%. For clarity, the 239 overlapping genes corresponded to 28.3% of the DEGs and 14.6% of the turquoise-module genes. These 239 genes were subsequently used as OA-associated candidate genes for intersection with PET-predicted targets.

### 3.3 Identification and functional enrichment of PET-associated candidate genes in OA

**FIGURE 4:**
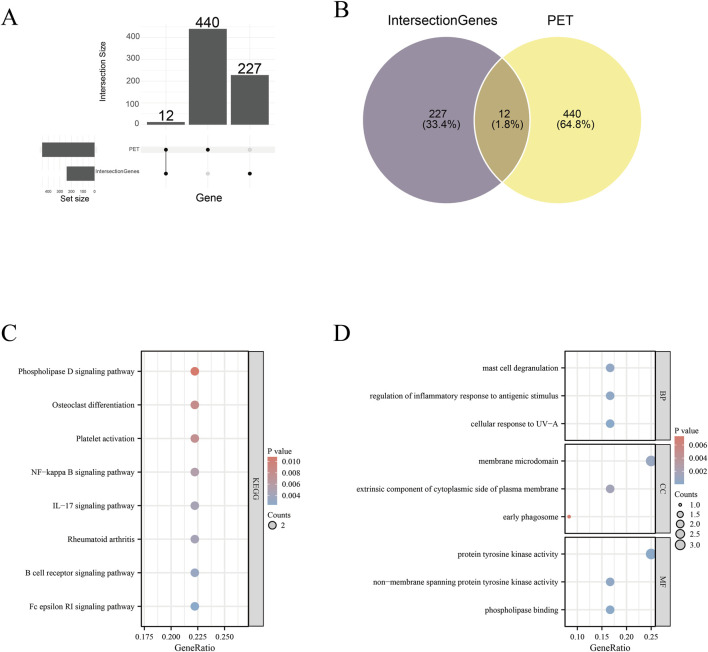
Identification and functional enrichment analysis of candidate genes shared between PET-associated targets and OA-related genes. **(A)** UpSet plot showing the intersection between the predicted PET-associated targets and OA-related candidate genes. Twelve genes were shared by the two gene sets. **(B)** Venn diagram illustrating the overlap between the two gene sets, including 12 shared candidate genes. **(C)** KEGG pathway enrichment analysis of the shared candidate genes. The enriched terms included the phospholipase D signaling pathway, osteoclast differentiation, platelet activation, NF-κB signaling pathway, IL-17 signaling pathway, rheumatoid arthritis, B-cell receptor signaling pathway, and Fc epsilon RI signaling pathway. **(D)** Gene Ontology enrichment analysis of the shared candidate genes across the biological process (BP), cellular component (CC), and molecular function (MF) categories. **(G)** UpSet plot showing the intersection size between DEGs and turquoise-module genes. **(H)** Venn diagram showing the overlap between DEGs and turquoise-module genes.

Intersection analysis of the predicted PET-associated targets and OA-related candidate genes identified 12 shared genes (Figure 4A,B). KEGG enrichment analysis linked these candidate genes to several signaling and disease-related terms, including the phospholipase D signaling pathway, osteoclast differentiation, platelet activation, NF-κB signaling pathway, IL-17 signaling pathway, rheumatoid arthritis, B-cell receptor signaling pathway, and Fc epsilon RI signaling pathway (Figure 4C). Gene Ontology analysis further associated the shared genes with biological processes such as mast cell degranulation, regulation of the inflammatory response to antigenic stimulus, and cellular response to UV-A. Enriched cellular-component terms included membrane microdomain, the extrinsic component of the cytoplasmic side of the plasma membrane, and early phagosome. The molecular-function terms included protein tyrosine kinase activity, non-membrane-spanning protein tyrosine kinase activity, and phospholipase binding (Figure 4D). These enrichment results indicate that the shared candidate genes are associated with inflammatory and signal-transduction annotations.

### 3.4 Identification of Core Genes in PET-Induced OA

**FIGURE 5:**
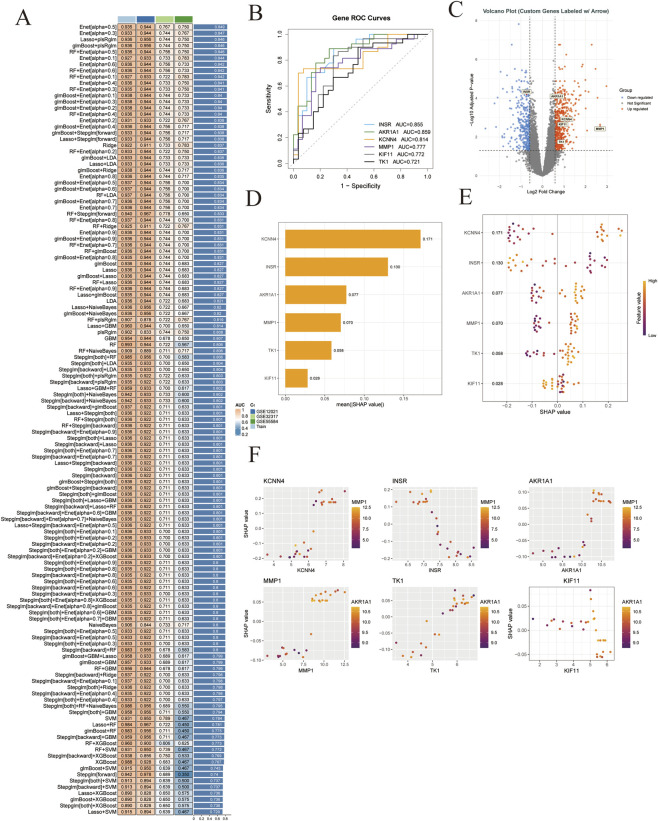
Identification of Core Genes in PET-MP-Induced OA. **(A)** Model Performance Comparison: A heatmap displays the AUC metrics of different models evaluated on multiple cohorts. The left section lists model names, while the right section presents corresponding AUC scores (higher values indicate superior performance). Color coding denotes the source of each cohort. **(B)** ROC Curve Analysis: Receiver operating characteristic curves illustrate the discriminative ability of selected genes (including AKR1A1, INSR, KIF11, MMP1, KCNN4, and TK1). The abscissa represents the false positive rate, and the ordinate corresponds to sensitivity. The area under the curve (AUC) serves as a measure of predictive accuracy. **(C)** Volcano Plot of Core Genes: A volcano plot visualizes differentially expressed genes, with the x-axis indicating the log_2_ fold change and the y-axis showing the negative logarithm of the p-value. Upregulated and downregulated genes are highlighted in red and blue, respectively, and key genes are explicitly labeled. **(D)** Feature Importance Ranking: A horizontal bar chart ranks the most influential genes based on their feature importance scores. Bar length reflects the magnitude of each gene’s contribution to model decisions. **(E)** Expression Distribution Visualization: Violin plots depict the distribution of gene expression under different experimental conditions. Plot width represents the density of observations, and color intensity reflects expression levels. **(F)** SHAP Value Distribution: Scatter plots display SHAP values for crucial genes, demonstrating their directional influence on model predictions.

Machine-learning analysis was performed to prioritize core genes among the 12 PET-associated OA candidate genes. The training cohort included 57 samples from GSE55235, GSE55457, and GSE82107, consisting of 30 OA samples and 27 controls. Three independent GEO datasets, including GSE12021, GSE32317, and GSE55584, were used as external validation cohorts, comprising 54 samples in total, with 25 OA samples and 29 controls. A total of 127 algorithm, parameter, and two-stage feature-selection/modeling combinations were evaluated (Figure 5A). Among these models, Enet[alpha=0.5] showed the best overall performance across the training and validation datasets. This model achieved AUC values of 0.935 in the training cohort and 0.944, 0.767, and 0.750 in GSE12021, GSE32317, and GSE55584, respectively. The Enet[alpha=0.5] model retained six genes, including AKR1A1, INSR, KIF11, MMP1, KCNN4, and TK1, which were therefore prioritized for downstream interpretation and experimental validation (Figure 5B).

The differential expression patterns of these six genes in OA tissues were visualized using a volcano plot (Figure 5C). SHAP analysis was then performed to interpret the relative contribution of each gene to the model output. KCNN4 (SHAP value = 0.171) and INSR (SHAP value = 0.130) showed the highest relative contributions among the six genes (Figure 5D). SHAP distribution and dependence analyses further illustrated how gene-expression variation was associated with model predictions (Figure 5E,F). These SHAP results were used to support candidate-gene prioritization and model interpretation, rather than to infer causal or mechanistic effects.

### 3.5 Molecular docking analysis of PET-Core protein interactions

**FIGURE 6:**
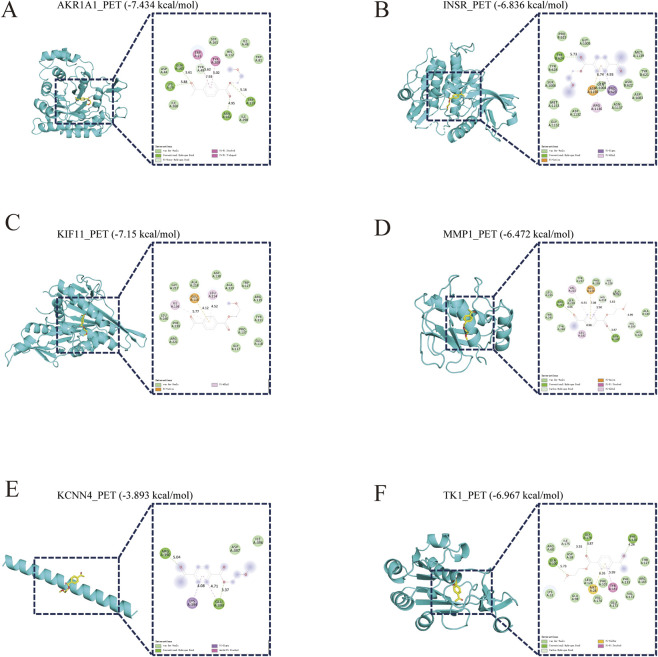
Molecular docking analysis of PET with six OA-related core proteins. **(A–F)** Structural visualization of PET binding with AKR1A1 **(A)**, INSR **(B)**, KIF11 **(C)**, MMP1 **(D)**, KCNN4 **(E)**, and TK1 **(F)**. For each protein, the 3D ribbon diagram illustrates the docking conformation of PET (yellow ligand), while the corresponding 2D interaction map highlights key amino acid residues involved in hydrogen bonding, hydrophobic interactions, and van der Waals forces. The binding affinities (in kcal/mol) were as follows: AKR1A1 (−7.434), KIF11 (−7.150), TK1 (−6.967), INSR (−6.836), MMP1 (−6.472), and KCNN4 (−3.893). More negative Vina scores indicate more favorable predicted docking scores in this computational analysis.

To explore the potential docking poses between PET and the screened core proteins, including AKR1A1, INSR, KIF11, MMP1, KCNN4, and TK1, molecular docking analyses were performed using AutoDock Vina. The predicted Vina scores were AKR1A1 (−7.434 kcal/mol), KIF11 (−7.150 kcal/mol), TK1 (−6.967 kcal/mol), MMP1 (−6.472 kcal/mol), INSR (−6.836 kcal/mol), and KCNN4 (−3.893 kcal/mol) (Table 1). Among these proteins, AKR1A1, KIF11, TK1, MMP1, and INSR showed relatively more negative predicted Vina scores, whereas KCNN4 showed the least favorable score. Therefore, KCNN4 was not interpreted as showing strong predicted binding, and its inclusion as a core gene was supported primarily by machine-learning feature importance and *in vitro* expression validation rather than by docking results. Visualization of the predicted docking conformations (Figure 6A–F) showed the computationally predicted PET poses and residue-level interaction patterns for each protein.

**TABLE 1:** Predicted binding energies of PET with six OA-Related core proteins (AutoDock vina).

Ligand	Receptor	Bind.Energy[kcal/mol]
PET	AKR1A1	−7.434
PET	INSR	−6.836
PET	KIF11	−7.150
PET	MMP1	−6.472
PET	KCNN4	−3.893
PET	TK1	−6.967

Residue-level interaction analysis was further performed for the top-ranked docking pose of each PET-core protein complex. PET interacted with the six core proteins through mixed non-covalent contacts, including hydrogen-bond contacts, carbon hydrogen bonds, van der Waals contacts, π-related hydrophobic contacts, and electrostatic π-cation/π-anion/π-sulfur interactions. AKR1A1 formed multiple predicted hydrogen-bond contacts with LYS79, TRP113, ASN162, and GLN183, together with π-π interactions involving TRP21 and TYR209. INSR showed one hydrogen-bond contact with TYR624 and additional π-cation, π-sigma, and π-alkyl contacts involving ASP1150, PRO623, and ARG1136. KCNN4 displayed fewer interactions, mainly involving ARG390, GLU393, and LYS394, consistent with its relatively weaker docking score. KIF11 was mainly associated with van der Waals and π-related/electrostatic contacts, whereas MMP1 and TK1 additionally showed hydrogen-bond contacts. Detailed residues, interaction types, interaction numbers, and distances are provided in Supplementary Table S2. These computational predictions suggest potential molecular interactions between PET and OA-related hub proteins.

### 3.6 *In vitro* experimental validation of core gene expression

**FIGURE 7:**
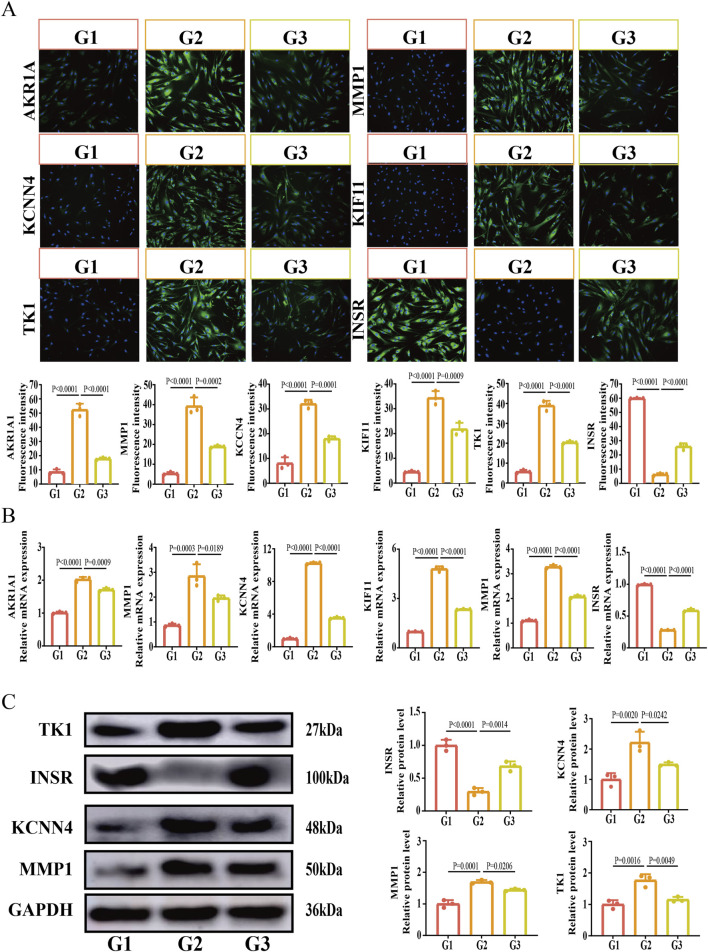
*In vitro* experimental validation of six core gene expression in PET-MP-treated chondrocytes. **(A)** Immunofluorescence staining of AKR1A1, MMP1, KCNN4, KIF11, TK1, and INSR in chondrocytes under three conditions: G1 (normal control), G2 (LPS+PET-MP treatment), and G3 (LPS stimulation). Representative fluorescence images are shown in the upper panels, with corresponding quantitative analysis of fluorescence intensity presented as bar graphs in the lower panels. **(B)** qRT-PCR analysis of relative mRNA expression levels of the six core genes across the three experimental groups. **(C)** Western blot analysis of protein expression levels for TK1, INSR, KCNN4, and MMP1.

To experimentally validate the bioinformatics predictions, we performed *in vitro* experiments using chondrocytes exposed to PET-MP. Three experimental conditions were established: normal control (G1), LPS+PET-MP treatment (G2), and LPS-induced inflammation (G3). Immunofluorescence staining showed coordinated dysregulation of the six core genes across the three groups (Figure 7A). LPS + PET-MP treatment significantly increased the fluorescence intensity of AKR1A1, MMP1, KCNN4, KIF11, and TK1 compared with the control group, whereas INSR fluorescence was significantly reduced. LPS alone induced directionally similar changes, but the signal intensities of most upregulated genes were lower than those observed in the LPS + PET-MP group. These findings support a PET-MP-associated amplification of inflammatory gene-expression changes in chondrocytes.

Consistent with the immunofluorescence findings, qRT-PCR confirmed coordinated dysregulation of the six core genes in treated chondrocytes (Figure 7B). Compared with the control group, LPS + PET-MP treatment significantly increased the mRNA expression of AKR1A1, MMP1, KCNN4, KIF11, and TK1, whereas INSR expression was significantly decreased. LPS alone induced directionally similar changes, but the magnitude of the upregulated genes was generally lower than that observed after combined LPS + PET-MP exposure. These results support a PET-MP-associated amplification of inflammatory gene dysregulation in chondrocytes.

Western blot analysis further confirmed the protein-level changes in four representative core genes (Figure 7C). TK1, KCNN4, and MMP1 protein expression was significantly increased after LPS + PET-MP treatment compared with the control group, whereas INSR expression was significantly decreased. LPS alone produced directionally similar changes, indicating that these proteins were also responsive to inflammatory stimulation. Together with the immunofluorescence and qRT-PCR results, these findings support the dysregulation of the core genes in treated chondrocytes.

## 4 Discussion

PET-MP have rapidly emerged as a new class of environmental pollutant that can reach articular cartilage through systemic circulation and the joint cavity’s synovial fluid. Emerging evidence positions microplastic pollution as a previously unrecognised environmental determinant of OA. Here, by integrating high-resolution transcriptomics, systems toxicology and molecular docking, we provide mechanistic insight into how PET-MP may erode articular cartilage homeostasis. Employing a combination of network toxicology and bioinformatic approaches, we identified 12 candidate genes. Among these, six hub genes—namely AKR1A1, INSR, KIF11, MMP1, TK1, and KCNN4—were distinguished as being of particular functional and mechanistic importance. Differential gene expression analysis revealed a consistent pattern of reduced INSR expression coupled with elevated transcript levels of AKR1A1, KIF11, MMP1, TK1, and KCNN4 in OA samples. These six high-confidence hub genes collectively map oxidative stress, metabolic reprogramming, senescence and extracellular-matrix (ECM) destruction. Molecular docking predicted energetically favourable interactions between PET oligomers and the corresponding target proteins, suggesting a potential ligand-like interaction mode.

Among the six hub genes, Aldo-keto reductase family 1 member A1 (AKR1A1) displayed the most favorable predicted binding energy with PET (−7.43 kcal mol^-1^) and relatively high SHAP importance (SHAP value = 0.077). This aldo-keto reductase detoxifies lipid aldehydes arising from peroxidation, thereby preserving membrane integrity (Barski et al., 2008). AKR1A1 governs key physiological and pathological processes. Recent work shows that AKR1A1 directs mesenchymal stem cell (MSC) fate toward adipogenesis or osteogenesis via a PKM2–PGC1α–SIRT1 axis (Chiang et al., 2025). Parallel studies in conditional AKR1A1-null mice presented with a reduction in ascorbic acid production and an increased level of ROS in the serum and, consequently, showed insufficient trabecular and cortical bone (Lai et al., 2017). These research on AKR1A1 in bone tissue offers a mechanistic explanation for the amplified oxidative injury observed in OA cartilage exposed to PET-MP. Insulin receptor (INSR) was bound PET-MP with −6.84 kcal mol^-1^and consistently downregulated in OA. Cartilage relies on INSR signalling to drive glucose uptake via GLUT4 and collagen II synthesis via PI3K-Akt-mTORC1 (Zhu et al., 2020). Our SHAP-based interpretation revealed a non-monotonic, U-shaped relationship between INSR expression and osteoarthritis (OA) risk. Both relatively low expression levels (< 7 log_2_TPM) and elevated expression (> 8 log_2_TPM) were associated with increased OA susceptibility. This biphasic pattern parallels the documented dual role of insulin resistance in OA development among diabetic populations with significant microplastic exposure. (Halabitska et al., 2024). Rosa et al. demonstrated that INSR expression in osteoarthritic cartilage falls to ≈40% of normal, a decline that blunts insulin-dependent glucose uptake yet preserves the hormone’s anabolic stimulation of type II collagen synthesis. This selective functional loss positions INSR deficiency as a metabolic driver of cartilage dysfunction in OA (Rosa et al., 2011). Complementing this observation, Halabitska et al. showed that systemic hyperinsulinaemia accelerates cartilage degeneration *via* over-activation of INSR-mediated PI3K/Akt-mTOR signalling. Conversely, metformin and GLP-1 analogues retard OA progression by lowering circulating insulin, restoring INSR sensitivity, and curbing mTOR-driven catabolism (Halabitska et al., 2024). These studies establish the insulin/INSR axis as both pathogenic linchpin and therapeutically tractable target: downregulation of INSR starves chondrocytes metabolically, while excessive ligand-dependent signalling erodes cartilage structure. Pharmacologic modulation of this axis—using agents such as metformin or GLP-1 analogues—thus emerges as a disease-modifying strategy for OA. kinesin family member 11 (KIF11), the plus-end kinesin-5 motor essential for bipolar spindle assembly (Yu et al., 2022), was markedly upregulated in osteoarthritic cartilage and exhibited high PET-MP binding affinity (−7.15 kcal mol^-1^). Single-cell RNA-seq data demonstrate a 2–3-fold enrichment of KIF11 in hypertrophic chondrocytes, coincident with primary cilium disassembly and dysregulated hedgehog signalling (Chen et al., 2018). Consistent with its mitotic role, over-expression of KIF11 causes spindle pole fragmentation, mitotic catastrophe, and chromosome congression defects (Dale et al., 2022). Collectively, these findings indicate that supraphysiological KIF11 levels disrupt chondrocyte homeostasis and may accelerate OA progression. MMP1 (Matrix Metallopeptidase 1) is the principal interstitial collagenase that cleaves native type-II collagen, the dominant tensile component of articular cartilage (Billinghurst et al., 1997). SHAP analysis revealed a biphasic contribution: moderate induction (5–6 log2TPM) promoted adaptive remodelling, whereas excessive levels (>10) signalled irreversible cartilage loss. Over-expression of MMP1, the principal collagenase that cleaves native type-II collagen, is now recognised as a key accelerator of OA (Milaras et al., 2021). Mechanistically, excess MMP1 degrades the collagen-II network, generating neo-epitopes that activate TLR4/NF-κB signalling. This amplifies IL-1β/TNF-α release, sustains synovial inflammation and further upregulates MMP1, creating a feed-forward loop that erodes cartilage tensile strength (Maouche et al., 2024). Concurrently, MMP1 released from osteoclasts disrupts subchondral bone plate integrity, facilitating trabecular micro-fractures that accelerate end-stage cartilage loss (Larrouture et al., 2021). Thus, MMP1 over-expression couples matrix degradation to inflammation and bone remodelling, validating MMP1 as a druggable node downstream of PET-MP exposure. Potassium Calcium-Activated Channel Subfamily N Member 4 (KCNN4) is a calcium-activated K^+^ channel that modulates membrane potential and Ca^2+^ signalling (Oliván-Viguera et al., 2013). Employing SHAP interpretability analysis, KCNN4 (with a SHAP value of 0.171) and INSR (0.130) were identified as the two most impactful influential predictors. KCNN4 over-expression in osteoarthritic cartilage potentiates osteoclast fusion, amplifies synovial inflammation and accelerates cartilage destruction via matrix metalloproteinase release (Kang et al., 2014). In chondrocytes, KCNN4 over-expression hyperpolarises the membrane, accelerates Ca^2+^ oscillations and increases the release of IL-6, MMP-13 and NO, thereby promoting cartilage catabolism (Segarra-Queralt et al., 2024). Selective blockade of KCNN4 with TRAM-34 or its genetic deletion curbs osteoclast fusion, dampens synovial inflammation and markedly lessens cartilage and bone destruction in mouse models of inflammatory arthritis (Kang et al., 2014). Thymidine kinase 1 (TK1) catalyzes the rate-limiting phosphorylation of thymidine for DNA synthesis; its expression is tightly cell-cycle-dependent (Bitter et al., 2020). PET-MP exposure upregulated TK1 (1.3 log2FC), presumably to sustain chondrocyte proliferation under oxidative stress. However, excessive TK1 activity exhausts intracellular NAD^+^ pools required for PARP-mediated DNA repair, precipitating an energy crisis (Cantó et al., 2015). Over the past few years, proteomic and transcriptomic screens have unexpectedly identified TK1 as a stress-responsive mediator in cartilage (Donnenfield et al., 2023). Molecular docking (−6.97 kcal mol^-1^) indicates favourable binding of PET dimer to the TK1 active-site loop, potentially enhancing substrate turnover.

To bridge the gap between computational predictions and biological relevance, we performed a series of *in vitro* experiments including immunofluorescence staining, qRT-PCR, and Western blot analysis in chondrocytes treated with PET-MP. The experimental results provided preliminary biological support for our bioinformatics findings. PET-MP exposure significantly upregulated AKR1A1, MMP1, KCNN4, KIF11, and TK1, while downregulating INSR, at both the mRNA and protein levels. These expression changes are directionally consistent with the patterns observed in the transcriptomic analysis of OA tissues, reinforcing the hypothesis that PET-MP can directly modulate key molecular targets in chondrocytes. Importantly, we included an LPS-stimulated group as a positive control to model classical inflammatory activation in chondrocytes. The LPS group exhibited expression changes that were qualitatively similar but quantitatively less pronounced than those induced by PET-MP for most upregulated genes, suggesting that PET-MP may exert more potent effects on these specific targets compared to conventional inflammatory stimuli alone. This observation is particularly noteworthy, as it implies that PET-MP may influence inflammatory and stress-related molecular programs beyond those captured by classical LPS-induced responses. The stronger induction of KCNN4 and AKR1A1 by PET-MP relative to LPS further supports the notion that PET-MP uniquely perturb calcium-activated potassium channel signaling and oxidative stress defense mechanisms, respectively, consistent with the high SHAP importance values assigned to these genes in our machine learning analysis.

These findings suggest that PET-MP may promote OA by interacting with key genes such as AKR1A1, INSR, KIF11, MMP1, KCNN4, and TK1. The convergence of bioinformatics predictions, molecular docking simulations, and *in vitro* experimental evidence provides a multi-layered framework for understanding how PET-MP may influence OA-related molecular pathways. These findings offer potential targets for the development of new therapeutic strategies and biomarkers for OA in the future.

Several limitations should be acknowledged. First, the bioinformatics analyses were based mainly on retrospective public GEO datasets, and the upstream candidate-gene selection was not nested within cross-validation, which may introduce optimistic bias in model performance estimates. Larger prospective cohorts with quantified PET-MP exposure and fully independent validation datasets are needed to confirm the robustness and generalizability of the identified markers. Second, the *in vitro* validation was performed using a single PET-MP concentration (2.4 μg/mL), a single LPS concentration (1 μg/mL), and a fixed exposure duration (24 h), which limits assessment of dose-response relationships and temporal dynamics. Third, the present study lacks *in vivo* validation in joint tissues; future animal models using intra-articular or systemic PET-MP exposure should be used to examine the tissue-specific localization and pathological roles of AKR1A1, INSR, KIF11, MMP1, KCNN4, and TK1. Finally, pathway enrichment and molecular docking results should be interpreted as hypothesis-generating evidence rather than direct proof of NF-κB pathway activation or PET-protein binding. Future studies should evaluate p65 phosphorylation and nuclear translocation, and further validate predicted PET-protein interactions using molecular dynamics simulations and experimental binding assays.

## 5 Conclusion

By combining multi-omics data with machine learning, molecular docking approaches, and *in vitro* experimental validation, this study establishes PET-MPs as a stressor relevant to OA, which modulates transcriptional networks in chondrocytes *via* six central genes. Each gene contributes a distinct yet interconnected facet—oxidative detoxification (AKR1A1), metabolic signalling (INSR), mitotic fidelity (KIF11), matrix remodelling (MMP1), Ca^2+^ homeostasis (KCNN4), and nucleotide metabolism (TK1)—culminating in cartilage destruction. The *in vitro* validation confirmed the directional consistency of expression changes predicted by bioinformatics, strengthening the translational relevance of our findings.

## Data Availability

The original contributions presented in the study are included in the article/Supplementary Material, further inquiries can be directed to the corresponding authors.
