# Supplementary material for: Integrating network toxicology and in vitro validation to elucidate PET microplastic-induced osteoarthritis pathogenesis
Source: Front Pharmacol. 2026 Jun 30;17:1859004. doi: 10.3389/fphar.2026.1859004 (PMC13365120; doi:10.3389/fphar.2026.1859004)
Supplement: Supplementary file 1 [file Supplementaryfile1.docx]

| **Target**  **(Gene)** | **PDB ID** | **Experimental method** | **Resolution**  **(Å)** | **R-value / R-free** | **Residue range** | **UniProt ID** | **Grid center**  **(x, y, z)** | **Grid size (x, y, z)**  **(Å)** | **Docking parameters** | **Control ligand** | **Replicates** |
| --- | --- | --- | --- | --- | --- | --- | --- | --- | --- | --- | --- |
| **AKR1A1** | 2ALR | X-ray | 2.48 | 0.190 / — | 1–324 | P14550 | (9.0, −2.3, 32.6) | (60.4, 66.7, 59.4) | Vina 1.2.7, exhaust=16, grid=0.375Å, 9 modes | Not used | 1 run |
| **INSR** | 3BU3 | X-ray | 1.65 | 0.202 / 0.220 | 987–1283 | P06213 | (6.5, 0.9, 21.7) | (57.9, 65.2, 65.7) | Vina 1.2.7, exhaust=16, grid=0.375Å, 9 modes | Not used | 1 run |
| **MMP1** | 1HFC | X-ray | 1.50 | 0.174 / — | 107–263 | P03956 | (25.1, 22.1, 24.3) | (22.0, 15.1, 15.8) | Vina 1.2.7, exhaust=16, grid=0.375Å, 9 modes | Not used | 1 run |
| **KCNN4** | 6D42 | X-ray | 1.75 | 0.252 / 0.323 | 376–414 | O15554 | (20.3, 7.1, 17.0) | (25.8, 24.2, 68.1) | Vina 1.2.7, exhaust=16, grid=0.375Å, 9 modes | Not used | 1 run |
| **KIF11** | 1X88 | X-ray | 1.80 | 0.203 / 0.225 | 18–364 | P52732 | (44.2, 29.0, 106.1) | (23.3, 27.6, 26.2) | Vina 1.2.7, exhaust=16, grid=0.375Å, 9 modes | Not used | 1 run |
| **TK1** | 1XBT | X-ray | 2.40 | 0.201 / 0.232 | 18–191 | P04183 | (54.8, 76.1, 3.5) | (22.2, 20.4, 16.4) | Vina 1.2.7, exhaust=16, grid=0.375Å, 9 modes | Not used | 1 run |

**Supplementary Table S1.** Protein crystal structures and molecular docking parameters.

*Note: All structures are from the Protein Data Bank (PDB).*

**Supplementary Table S2.** Residue-level interaction profiles of PET with the six core proteins based on the top-ranked AutoDock Vina docking poses.

| **Complex** | **Vina score (kcal/mol)** | **Hydrogen-bond contacts and distances** | **Hydrophobic / π-related contacts** | **van der Waals residues** | **Electrostatic contacts** | **Dominant contributing residues** | **Quantitative summary** |
| --- | --- | --- | --- | --- | --- | --- | --- |
| PET-AKR1A1 | -7.434 | 4: LYS79 (5.88 Å), TRP113 (5.16 Å), ASN162 (4.95 Å), GLN183 (5.61 Å) | 2 π-π contacts: TRP21 (5.62 Å), TYR209 (5.02 Å); 1 π-donor H-bond contact: TYR49 (7.93 Å) | ASP44, ILE48, HIS112, SER161, ILE260, ILE298 | Not detected in the 2D interaction map | LYS79, TRP113, ASN162, GLN183, TRP21, TYR209 | Hydrogen-bond contacts: 4; π-related contacts: 3; van der Waals residues: 6 |
| PET-INSR | -6.836 | 1: TYR624 (5.73 Å) | 1 π-sigma contact: PRO623 (4.93 Å); 1 π-alkyl contact: ARG1136 (5.74 Å) | GLY1005, SER1006, PRO625, TYR628, TYR621, ASN622, MET1139, ASN1137, ASP1083, MET1153, ASP1132, GLY1152 | 1 π-cation contact: ASP1150 (6.74 Å) | TYR624, ASP1150, PRO623, ARG1136 | Hydrogen-bond contacts: 1; π-related/electrostatic contacts: 3; van der Waals residues: 12 |
| PET-KCNN4 | -3.893 | 2: ARG390 (5.04 Å), GLU393 (3.37 Å) | 1 π-sigma contact: LYS394 (4.08 Å); 1 amide-π stacked contact: LYS394 (4.71 Å) | ILE396, ASP397 | Not detected in the 2D interaction map | ARG390, GLU393, LYS394 | Hydrogen-bond contacts: 2; π-related contacts: 2; van der Waals residues: 2 |
| PET-KIF11 | -7.150 | No conventional hydrogen bond detected in the 2D interaction map | 2 π-alkyl contacts: ILE136 (5.77 Å), LEU214 (4.52 Å) | GLY217, ALA218, ASP130, ALA133, TRP127, ARG119, TYR211, PRO137, GLU118, GLY117, LEU160, PHE239, ARG221 | 1 π-cation contact: GLU116 (4.12 Å) | GLU116, ILE136, LEU214 | Hydrogen-bond contacts: 0; π-related/electrostatic contacts: 3; van der Waals residues: 13 |
| PET-MMP1 | -6.472 | 2 conventional H-bonds: ARG214 (4.66 Å), ASN180 (3.87 Å); 2 carbon H-bonds: HIS228 (5.63 Å), HIS183 (4.99 Å) | 1 π-π stacked contact: HIS218 (5.90 Å); 2 π-alkyl contacts: VAL215 (6.41 Å), LEU181 (4.98 Å) | LEU235, TYR237, PRO238, SER239, THR241, TYR240, ALA182, ALA184, HIS222 | 1 π-anion contact: GLU219 (7.08 Å) | ARG214, ASN180, GLU219, HIS218, VAL215, LEU181 | Hydrogen-bond contacts: 4; π-related/electrostatic contacts: 4; van der Waals residues: 9 |
| PET-TK1 | -6.967 | 3 conventional H-bonds: GLN100 (5.73 Å), GLY176 (3.87 Å), PHE128 (4.24 Å); 1 carbon H-bond: ILE175 (3.55 Å) | 1 π-π stacked contact: TYR181 (5.99 Å) | ARG60, ASP58, LYS32, GLU98, LEU124, PHE101, PHE133, THR127, ARG165, VAL172, GLU173, VAL174 | 1 π-sulfur contact: MET28 (6.35 Å) | GLN100, GLY176, PHE128, ILE175, MET28, TYR181 | Hydrogen-bond contacts: 4; π-related/electrostatic contacts: 2; van der Waals residues: 12 |

*Note: Interaction types and distances were extracted from the Discovery Studio-style 2D interaction maps in the docking result figures. The distances are reported in Å.*

**Supplementary Table S3.** Primer sequences used for quantitative real-time PCR (qRT-PCR).

| **Gene** | **Forward Primer (5′→3′)** | **Reverse Primer (5′→3′)** | **Amplicon (bp)** | **Annealing Temp. (°C)** |
| --- | --- | --- | --- | --- |
| **AKR1A1** | AGGTCCAGCGGAAAGTGATC | TGTTCAGGGCATTTAGCTGC | 123 | 60 |
| **MMP1** | ATGAAGCAGCCCAGATGTGGAG | TGGTCCACATCTGCTCTTGGCAA | 137 | 60 |
| **KCNN4** | CATTCCTGACCATCGGCTATGG | GCCTTGTTAAACTCCAGCTTCCG | 142 | 60 |
| **KIF11** | GATGGACGTAAGGCAGCTCA | TGTGGTGTCGTACCTGTTGG | 185 | 60 |
| **TK1** | AGCAGCTTCTGCACACATGACC | CTCGCAGAACTCCACGATGTCA | 144 | 60 |
| **INSR** | CTTCACCTGCCATCACGTGGTG | ATTCTCAGCCTCTGGCCGCAGA | 133 | 60 |
| **GAPDH** | CATTGCCCTCAACGACCACT | TCCTTGGAGGCCATGTGGGC | 105 | 60 |

**Supplementary Table S4.** Primary and secondary antibodies used for Western blot analysis.

| **Antibody** | **Antibody type** | **Host Species** | **Dilution** | **Manufacturer** | **Catalog No.** |
| --- | --- | --- | --- | --- | --- |
| **TK1** | Primary | Rabbit | 1:1000 | Proteintech (Wuhan, China) | 15691-1-AP |
| **INSR** | Primary | Rabbit | 1:1000 | Proteintech (Wuhan, China) | 20433-1-AP |
| **KCNN4** | Primary | Rabbit | 1:1000 | Proteintech (Wuhan, China) | 23271-1-AP |
| **MMP1** | Primary | Rabbit | 1:1000 | Proteintech (Wuhan, China) | 10371-2-AP |
| **GAPDH** | Primary | Rabbit | 1:10000 | Proteintech (Wuhan, China) | 10494-1-AP |
| **HRP-conjugated Goat Anti-Rabbit IgG (H+L)** | Secondary | Goat | 1:5000 | Proteintech (Wuhan, China) | SA00001-2 |

*Note: All primary antibodies were rabbit polyclonal antibodies raised against the corresponding human protein and were validated for Western blot by the manufacturer (Proteintech Group, Wuhan, China; www.ptgcn.com). GAPDH was used as the internal loading control.*
